# Involving patients in optimising RCT participant information sheets and exploring patient acceptability of clinical trials

**DOI:** 10.1186/1745-6215-14-S1-O71

**Published:** 2013-11-29

**Authors:** Alba Realpe, Peter Wall, Damian Griffin, Rachel Hobson, Jenny Donovan, Ann Adams

**Affiliations:** 1 Warwick Medical School, University of Warwick, Coventry, UK; 2 School of social and community medicine, University of Bristol, Bristol, UK

## 

Pragmatic multi-centre RCTs are acknowledged to be the best design for evaluating the effectiveness of health care interventions as they provide robust evidence of effect, but they often encounter recruitment difficulties. Randomised Controlled Trials (RCTs) in surgery require patients to accept uncertainty or equipoise between surgical and non-surgical treatments as well as to achieve a good understanding of the trial procedures. A qualitative study was conducted to explore the acceptability amongst patients of a RCT comparing hip arthroscopy for femoroacetabular impingement (FAI) versus nonoperative treatment and to help improve trial participant information for a subsequent pilot RCT. Eighteen patients who had been diagnosed with FAI and had received either surgery (N=13) or nonoperative treatment (N=5) were interviewed about their treatment experiences. They were also asked to evaluate the adequacy and readability of the participant information sheets. Results suggested that patients who are better informed about their health condition and possible treatments are more able to accept uncertainty amongst clinicians than those with limited knowledge. Patients were more inclined to accept randomisation if i. they felt the treatment was individualised to their needs, and ii. the same clinician would provide care. The findings of the study helped to brand the nonoperative intervention arm in a user-friendly way. The current recruitment rate of the pilot RCT is 70%. Collaborating with former patients in developing and optimising patient information sheet and exploring the acceptability of trials could contribute to improving informed consent and recruitment rates in future RCT.

